# The impact of different exercise modalities on chronic kidney disease: an umbrella review of meta-analyses

**DOI:** 10.3389/fphys.2024.1444976

**Published:** 2025-01-06

**Authors:** Hugo L. Correa, Thiago S. Rosa, Rafael L. Santos, Vitoria M. Mestrinho, Thaís S. Aquino, Weberth O. Santos, Rodrigo P. Neves, Lysleine A. Deus, Andrea L. Reis, Jessica M. Barbosa, Thais B. Araujo, Ruchama Verhoeff, Karim Yatim, Daniel Mendes, Roberto C. Manfro, Thiago J. Borges, Leonardo V. Riella

**Affiliations:** ^1^ Center for Transplantation Sciences, Department of Surgery, Massachusetts General Hospital, Harvard Medical School, Charlestown, MA, United States; ^2^ Graduate Program in Physical Education, Catholic University of Brasilia, Brasília, Brazil; ^3^ Graduate Program in Genomic Sciences and Biotechnology, Catholic University of Brasilia, Brasília, Brazil; ^4^ Department of Medicine, Catholic University of Brasilia, Brasília, Brazil; ^5^ Hospital de Clínicas de Porto Alegre, Porto Alegre, Rio Grande do Sul, RS – Brasil

**Keywords:** chronic kidney disease, exercise modalities, aerobic exercise, resistance training, combined training, eletromyostimulation, home-based exercise, meta-analysis

## Abstract

**Introduction:**

Exercise is widely recognized for its benefits to chronic kidney disease (CKD) patients. However, the specific impact of different exercise modalities on CKD-related outcomes remains unclear. This study sought to summarize the effects of different exercise modalities on the main outcomes impacted by CKD.

**Methods:**

We searched for systematic review with meta-analysis in PubMed, Embase, Web of Science, Scopus, and Cochrane databases. We evaluated the methodological quality of included studies by AMSTAR2 tool and by individually evaluating the heterogeneity, sample power, and statistical significances from meta-analyses.

**Results:**

We included 44 meta-analyses, encompassing 35,432 CKD patients in pre-dialysis and dialysis stages (peritoneal and hemodialysis). Data from meta-analyses with highly suggestive or strong evidence grading suggests that aerobic and combined training were most effective in improving cardiorespiratory fitness (main effect: 2.1, 95% CI: 0.8–3.4, and main effect: 3.4; 95% CI: 2.4–4.6, respectively). Combined training showed a consistent benefit in psychosocial domains (main effect: −7.3; 95% CI: −9.31 to −53). All exercise modalities significantly improve functional performance, except isometric training, which impacted just fistula maturation (main effect: 0.84; 95% CI: 0.5–1.2).

**Conclusion:**

Exercise emerges as a potential non-pharmacological therapy for CKD patients. Tailoring exercise to specific outcomes appears to be crucial, as different exercise modalities exhibit varying effectiveness.

## 1 Introduction

Chronic kidney disease (CKD) poses a growing global health challenge, impacting individuals and healthcare systems worldwide (Kovesdy, 2022; August, 2023). More than 800 million subjects are estimated to have CKD, reflecting the widespread prevalence of this condition (Jha et al., 2023; KDIGO, 2020, 2020). The progressive decline in kidney function associated with CKD leads to a range of complications, from diminished quality of life to major cardiovascular events (Jha et al., 2023; Tsai et al., 2015). To mitigate these deleterious effects, CKD patients often require several pharmacological interventions (de Boer et al., 2022; Navaneethan et al., 2023; Dong et al., 2022; Fan et al., 2022). While pharmaceutical treatments are essential to CKD management, they are frequently complemented by non-pharmacological approaches to address the nature of this disease (Rovin et al., 2021; Bishop et al., 2023; Johansen and Painter, 2012). Exercise has emerged as a promising non-pharmacological intervention for CKD, as it appears to improve outcomes of CKD patients, without reported side effects (Sprick et al., 2022; Corrêa et al., 2021a; Beetham et al., 2022; de Araújo et al., 2023; Gadelha et al., 2021; Corrêa et al., 2021b; Deus et al., 2022).

Recent studies have showcased its potential to enhance both physical and psychological health parameters (Deus et al., 2021). Many systematic reviews and meta-analyses have consolidated the role of exercise in improving CKD-related outcomes (Andrade et al., 2022; Andrade et al., 2019; Baião et al., 2023; Bernier-Jean et al., 2022; Bogataj et al., 2019; Burrai et al., 2021; Cai et al., 2022; Cheema et al., 2014; Chung et al., 2017; Clarkson et al., 2019; Ferrari et al., 2020; Ferreira et al., 2019; Ferreira et al., 2021; Gomes Neto et al., 2018; Hargrove et al., 2021; Huang et al., 2019; Junqué-Jiménez et al., 2022; Liu et al., 2022; Ma et al., 2022; Matsuzawa et al., 2017; Meng et al., 2022; Nakamura et al., 2020; Nantakool et al., 2022; Nantakool et al., 2020; Pei et al., 2019; Pu et al., 2019; Qiu et al., 2017; Sawant et al., 2014; Scapini et al., 2019; Schardong et al., 2020; Sheng et al., 2014; Smart and Steele, 2011; Song et al., 2022; Song et al., 2018; Thompson et al., 2019; Vanden Wyngaert et al., 2018; Villanego et al., 2020; Wang et al., 2022; Wu et al., 2021; Wu et al., 2020; Yang et al., 2017; Yang et al., 2020; Young et al., 2018; Zhang et al., 2019), leading major international kidney disease organizations to endorse its inclusion in CKD management (Milam, 2016). While two umbrella reviews have demonstrated the comprehensive positive impact of exercise (Zhang et al., 2022) and resistance training (Perez-Dominguez et al., 2023) on variables such as blood pressure, muscle strength, body composition, dialysis-related symptoms, and quality of life, a comprehensive understanding of the collective influence of different exercise modalities on CKD-related outcomes remains unclear. Prior reviews often focus on specific modalities or isolated outcomes, leaving gaps in the broader understanding of how different exercise approaches impact a range of clinically relevant outcomes. The variability in study designs and populations across reviews poses challenges in consolidating findings into actionable insights. Covering a wide range of outcomes, including blood pressure, body composition, functional performance, and quality of life is relevant for understanding the impact of exercise on CKD (Zhang et al., 2022; Perez-Dominguez et al., 2023).

A deeper understanding of how specific exercise modalities influence CKD-related outcomes is critical for refining clinical guidelines (Rovin et al., 2021; Bishop et al., 2023; Johansen and Painter, 2012; Sprick et al., 2022). Tailored exercise prescriptions may optimize benefits such as improving cardiorespiratory fitness, mitigating inflammation, and enhancing quality of life (Corrêa et al., 2021a; Gadelha et al., 2021; Ferrari et al., 2020; Pei et al., 2019). Aerobic training has demonstrated significant improvements in inflammatory markers and cardiorespiratory fitness, contributing to better cardiovascular health (Ferrari et al., 2020; Pei et al., 2019; Diniz et al., 2021). Resistance training is essential for enhancing muscle strength and functional performance, addressing the prevalent issue of sarcopenia in CKD patients (Corrêa et al., 2021a; Gadelha et al., 2021; Schoenfeld, 2010). Combined training may provide synergistic benefits by targeting both cardiorespiratory and musculoskeletal outcomes (Burrai et al., 2021; Chung et al., 2017).

In addition to these well-studied approaches, other modalities such as isometric, home-based, and respiratory exercises have emerged as practical alternatives that may overcome barriers to exercise adherence, particularly for patients with limited mobility or access to structured programs (Nantakool et al., 2022; Liu-Ambrose et al., 2019). Electromyostimulation (EMS) offers a novel avenue for improving muscle strength in patients unable to engage in traditional exercise, while respiratory training may target specific deficits in lung function and overall endurance (Ferrari et al., 2020; Song et al., 2022). These modalities, though less explored, hold promise for expanding the scope of CKD management and addressing unique patient needs. Exploring these approaches collectively is crucial for bridging gaps in current evidence and tailoring interventions for clinical practice.

To fill this gap, this umbrella review sought to summarize the impact of different exercise modalities on specific CKD-related outcomes: spanning blood pressure, body composition, cardiorespiratory fitness, clinical routine parameters, functional performance, inflammatory markers, kidney function, psychosocial domains, quality of life, sleep quality, vascular function, and mortality. Within this framework, we explore the ramifications of aerobic, resistance, combined, eletromyostimulation (EMS), isometric, home-based, and respiratory training in CKD. This exploration reveals that each modality may exhibit varying degrees of effectiveness for specific outcomes, underscoring the importance of customizing exercise regimens for this population.

## 2 Methods

### 2.1 Protocol and registration

We performed an umbrella-review according to the Cochrane Handbook recommendations (Higgins and Green, 2008) and reported the results according to the preferred reporting items for overviews of reviews (PRIOR) statement (Gates et al., 2022). The protocol for this review was registered in the International Prospective Register of Systematic Reviews https://www.crd.york.ac.uk/prospero/#recordDetails with registration number: CRD42022381825.

### 2.2 Criteria for considering studies for this review

Eligible studies for this umbrella review were systematic reviews with meta-analyses published in peer-reviewed journals. The population of interest included adults diagnosed with CKD at any stage. Studies were considered if they evaluated chronic exercise interventions, including aerobic, resistance, combined, isometric, EMS, home-based, or respiratory training, compared to usual care, no intervention, or other exercise modalities. Eligible outcomes included physiological parameters (e.g., blood pressure, kidney function), physical performance (e.g., muscle strength, cardiorespiratory fitness), and psychosocial factors (e.g., quality of life, sleep quality). There were no restrictions on language, age, sex, or disease stage. Exercise training regimens were categorized by modality where specified, and studies with unspecified regimens were classified as “isotonic” training for consistency. Studies were excluded if they did not include a meta-analytical component or if the population was non-CKD or pediatric.

### 2.3 Search and selection strategy

Two authors (TM and RL) independently reviewed published meta-analytic systematic reviews by searching PubMed, Scopus, Web of Science, and the Cochrane Library from their inception to December 2023. All searches were adapted from the MEDLINE search strategy as reported: (“Exercise training” [MeSH]) AND (“Chronic Kidney Disease” [MeSH). We reviewed the trials’ bibliographies, identifying and contacting some of the authors in the field to clarify trial eligibility or to identify additional published or unpublished data. Next, two review authors (TM and RL) independently checked the references identified by the search strategy. The full texts of all potentially relevant studies were obtained for independent assessment. Disagreements were solved through discussion, and a third review author (HLC) acted as arbitrator where necessary. All citations were downloaded into EndNote X9^®^, duplicates were removed, and an identification number was assigned to each article.

### 2.4 Data extraction

The same authors collected data in sufficient detail to better extract properties including studies based on PICO: Population: CKD patients; Intervention: exercise training; Comparator, no exercise group; Outcome: CKD-related outcomes. We also extracted Statistical aspects such as *P*-value, heterogeneity (I^2^), main effect, and largest study effect were also extracted. Data was collected from text, tables, and figures. After extracting the data, two authors (TM and RL) graded the risk of bias in the included trials. Disagreements were resolved through discussion and a third reviewer (HLC) acted as moderator where necessary. Authors of primary studies did not extract data from their own studies. RL entered the data into the Excell (Microsoft Corporation) and HLC checked data entry. We calculated the corrected covered area (CCA) to assess the overlap of primary studies. The CCA accounts for the number of times primary studies are included across different reviews, providing a value that ranges from 0 (no overlap) to 1 (complete overlap). All graphs and tables in the present study were created using R and RStudio version 4.3.2.

### 2.5 Assess of risk of bias in included studies

The risk of bias for eligible studies was assessed using the AMSTAR 2 tool (Shea et al., 2017), which evaluates the methodological quality of systematic reviews and meta-analyses. The tool consists of 16 items, each scored as “yes,” “partial yes,” or “no.” Scoring percentages determined quality classification (high, medium, or low). The risk of bias assessment was conducted independently by two authors, with disagreements resolved by a third author. Two authors performed the risk of bias assessment independently using the AMSTAR 2 tool (Shea et al., 2017), which evaluates the methodological quality of systematic reviews and meta-analyses. This checklist contains 16 items, and each item was answered with a “yes” (1 point), “partial yes” (0.5 points) or “no” (0 points). The percentage score for each study was calculated using the total score as the numerator and the highest score of 16 points as the denominator. A meta-analysis scoring ≥80% was classified as high quality, 40%–79% as medium quality and those scoring <40% as low quality. Disagreements were solved by a third author (HLC).

### 2.6 Statistical methodology

This umbrella review synthesized evidence from included meta-analyses by evaluating methodological quality, heterogeneity, and consistency across studies. Heterogeneity was assessed using the I^2^ statistic, with thresholds of <25%, 25%–50%, and >50% indicating low, moderate, and high heterogeneity, respectively. Random-effects models were applied to account for variability in study populations and methodologies. The grading of evidence relied on effect size estimates, confidence intervals, and sample size, as defined in the grading framework. Statistical significance thresholds were based on predefined p-values, and heterogeneity levels informed the strength of evidence. Meta-analyses with inconsistent findings or significant heterogeneity were noted but not excluded, to provide a comprehensive synthesis of available evidence. No additional tests for publication bias or excessive significance bias were conducted. All graphs and tables in the present study were created using R and RStudio version 4.3.2.

### 2.7 Grading evidence

The grading of evidence for all included meta-analyses was established through an adapted framework, as previously described (Bellou et al., 2018; Papadimitriou et al., 2021; Botelho et al., 2022). This framework categorized evidence into four levels: strong, highly suggestive, suggestive, and weak. These levels were determined by thresholds for sample size, significance level, and heterogeneity. Strong evidence was attributed to meta-analyses with a sample size of more than 385 participants, a p-value ≤10^−^⁶, and low heterogeneity (I^2^ <50%). Highly suggestive evidence was assigned to meta-analyses with more than 385 participants, a random-effects p-value ≤10^−^⁶, and a significant effect reported in the largest contributing study. Suggestive evidence was applied to meta-analyses with more than 385 participants and a p-value ≤10^−^³. Weak evidence included meta-analyses that did not meet the above criteria. The threshold of 385 participants was chosen to ensure a 95% confidence level in estimates based on the global prevalence of CKD, which affects approximately 800 million individuals worldwide (Kovesdy, 2022; Kadam and Bhalerao, 2010).

## 3 Results

### 3.1 Characteristics of the included meta-analysis

**FIGURE 1:**
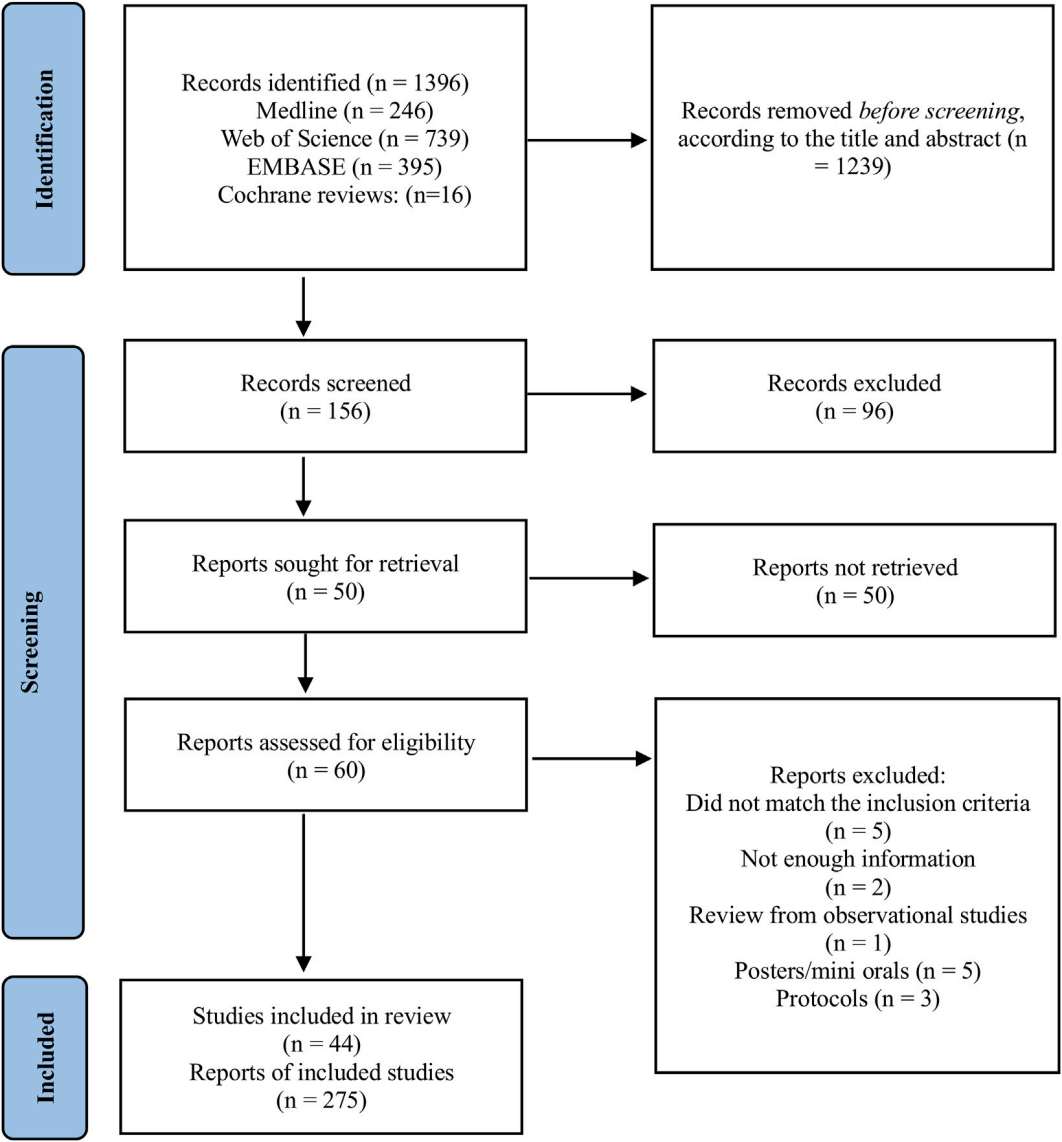
Flow chart diagram for study selection.

A total of 1,396 records were identified through database searches, including Medline (*n* = 246), Web of Science (*n* = 739), EMBASE (*n* = 395), and Cochrane Reviews (*n* = 16). After screening titles and abstracts, 1,239 records were excluded, leaving 156 records for further screening. Of these, 96 records were excluded based on inclusion and exclusion criteria applied during full-text screening. Following this, 60 reports were assessed for eligibility. Specific reasons for exclusions included reports that did not meet the inclusion criteria for study design or population (*n* = 5), lacked sufficient information for evaluation (*n* = 2), reviewed observational studies (*n* = 1), posters or mini-oral presentations without sufficient detail (*n* = 5), and protocols without completed analyses (*n* = 3). In total, we included 44 meta-analyses comprising data from 839 randomized controlled trials involving 35,431 patients with CKD, with patient numbers ranging from 124 to 3,846, encompassing both non-dialysis (stage 1–4) and dialysis patients. Each study was treated as an independent report according to the number of exercise modalities and interventions analyzed, resulting in a total of 275 reports (Figure 1). While duration of exercise interventions encompassed chronic exercise regimens (ranging from 4 to 24 weeks), it was not consistently reported between studies which limited detailed analysis on the impact of duration on our studied outcomes. The overall CCA for all exercise modalities was calculated as 0.46, reflecting a low to moderate overlap of primary studies. When stratifying intervention type the overlap varied. Isometric training and resistance training displayed a moderate overlap (0.5 and 0.6, respectively). Aerobic training presented a low overlap of primary studies (0.14). Combined and respiratory training presented a CCA >0.6 suggesting a moderate to high overlap.

Detailed characteristics of the included studies are provided in Table 1. Furthermore, we present detailed information on the included studies, describing each variable and the measurements used in Supplementary Tables S1, S2, respectively.

**TABLE 1:** Characteristics of the included studies.

Author	Disease stage	no studies	Sample size	Intervention	Outcomes
Nantakool et al. (2022)	Dialysis	9	526	Isotonic and isometric training	Fistula maturation
Ma et al. (2022)	Non-dialysis	11	166	Aerobic training	Kidney function
Song et al. (2022)	Dialysis	46	1893	Aerobic, resistance, EMS, combined, and resistance training	Functional performance, ktv, blood pressure
Andrade et al. (2022)	Dialysis	4	215	Isotonic training	Fistula maturation
Junqué-Jiménez et al. (2022)	Both	8	621	Home-based training	Functional performance, quality of life, and psychosocial (depressive symptoms)
Wang et al. (2022)	Both	18	817	Isotonic training	Vascular function
Wu et al. (2021)	Non-dialysis	16	724	Isotonic training	Inflammatory profile (IL6 and C-reactive protein) and body composition
Cai et al. (2022)	Dialysis	7	331	Combined training	Urea clearance and quality of life
Liu et al. (2022)	Dialysis	8	610	Isotonic training	Psychosocial (cognitive function)
Meng et al. (2022)	Dialysis	9	302	Isotonic training	Fistula maturation
Burrai et al. (2021)	Dialysis	5	462	Isotonic training	Functional performance
Ferreira et al. (2021)	Both	6	282	Combined training	Psychosocial (depressive and anxiety symptoms)
Hargrove et al. (2021)	Dialysis	15	108	Aerobic training	Psychosocial (depressive symptoms)
Nakamura et al. (2020)	Non-dialysis	18	848	Isotonic and home-based training	All-cause mortality and kidney function
Natankool et al. (2020)	Dialysis	5	413	Isotonic and isometric training	Fistula maturation and functional performance
Wu et al. (2020)	Non-dialysis	12	745	Isotonic training	Kidney function
Yang et al. (2021)	Non-dialysis	11	623	Isotonic training	Kidney function-related parameters (albumin and proteinuria)
Ferrari et al. (2020)	Both	50	1757	Aerobic, resistance, respiratory, and combined	Clinical routine parameters, cardiorespiratory fitness, functional performance, inflammatory markers (IL6 and CRP), and blood pressure
Schardong et al. (2020)	Dialysis	10	242	EMS training	Functional performance and quality of life
Bogataj et al. (2019)	Dialysis	33	1,274	Isotonic exercise	Functional performance
Andrade et al. (2019)	Dialysis	7	124	Aerobic and combined training	Cardiorespiratory fitness
Ferreira et al. (2019)	Dialysis	23	346	Isotonic training	Clinical routine parameters (ktv, creatinine)
Huang et al. (2019)	Dialysis	19	677	Isotonic, aerobic, combined and resistance training	Blood pressure, clinical routine parameters (ktv), cardiorespiratory fitness, functional performance, and quality of life
Thompson et al. (2019)	Dialysis	12	505	Isotonic training	Blood pressure
Scapini et al. (2019)	Dialysis	33	1,254	Aerobic, combined, and resistance training	Cardiorespiratory fitness, blood pressure, clinical routine parameters (ktv)
Pu et al. (2019)	Dialysis	27	1,215	Isotonic training	Clinical routine parameters, cardiorespiratory fitness, psychosocial (depressive symptoms), quality of life, blood pressure, and functional performance
Vanden Wyngaert et al. (2018)	Non-dialysis	11	382	Isotonic training	Kidney function, blood pressure, and cardiorespiratory fitness
Gomes Neto et al. (2018)	Dialysis	56	2,586	Aerobic, resistance, and combined	Cardiorespiratory fitness, functional performance, quality of life, and depressive symptoms
Young et al. (2018)	Dialysis	13	369	Aerobic training	Cardiorespiratory fitness, functional performance, quality of life, blood pressure, and vascular function
Song et al. (2018)	Dialysis	15	683	Isotonic training	Sleep quality and psychosocial (depressive symptoms and fatigue)
Chung et al. (2017)	Dialysis	17	651	Isotonic training	Functional performance, cardiorespiratory fitness, clinical routine parameters, psychosocial, and quality of life
Qiu et al. (2017)	Dialysis	9	255	Isotonic training	Blood pressure and cardiorespiratory fitness
Cheema et al. (2014)	Both	7	271	Isotonic training	Functional performance, body composition, and quality of life
Sheng et al. (2014)	Both	24	997	Isotonic training	Clinical routine parameters, cardiorespiratory fitness, and quality of life
Smart and Steele (2011)	Both	15	565	Isotonic training	Cardiorespiratory fitness
Villanego et al. (2020)	Non-dialysis	21	429	Combined training	Kidney function, body composition, blood pressure, cardiorespiratory fitness, and functional performance
Pei et al. (2019)	both	31	1,305	Aerobic training	Cardiorespiratory fitness, functional performance, clinical routine parameters, quality of life
Clarkson et al. (2019)	Dialysis	27	1,156	Isotonic, resistance, aerobic, respiratory, and combined training	Functional performance
Matsuzawa et al. (2017)	Dialysis	30	582	Isotonic training	Cardiorespiratory fitness, functional performance, quality of life
Yang et al. (2017)	Non-dialysis	5	179	Isotonic training	Cardiorespiratory fitness
Sawant et al. (2014)	Dialysis	5	206	Isotonic training	Functional performance
Zhang et al. (2019)	Non-dialysis	13	421	Isotonic training	Kidney function and blood pressure
Bernier-jean et al. (2022)	Dialysis	89	1,608	Isotonic training	All-cause mortality
Baião et al. (2023)	Both	29	2,834	Isotonic, resistance, aerobic, and combined training	Inflammatory profile (IL6, TNF, IL10)

### 3.2 Risk of bias assessment

**FIGURE 2:**
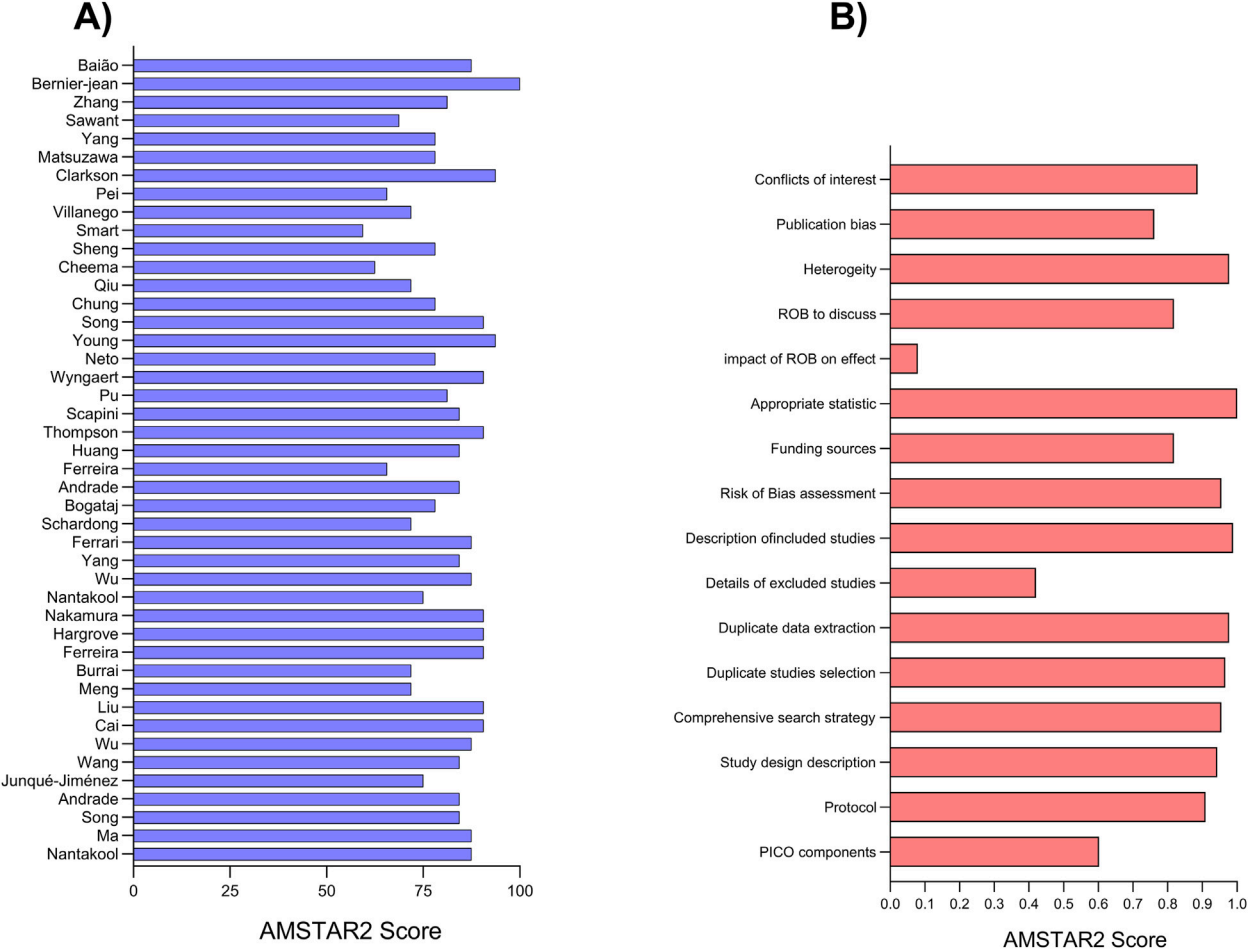
Risk of bias assessment by AMSTAR2+ according to each study **(A)** and items **(B)**.

The mean risk of bias score across all studies was 13 (equivalent to 81.61%), with scores ranging from a minimum of 9.5 (59.38%) to a maximum of 16 (100%). Notably, most studies received scores above 13, indicating a low risk of bias, with over 80% of studies surpassing this threshold. Described in Figure 2.

### 3.3 Trends in publications over time

**FIGURE 3:**
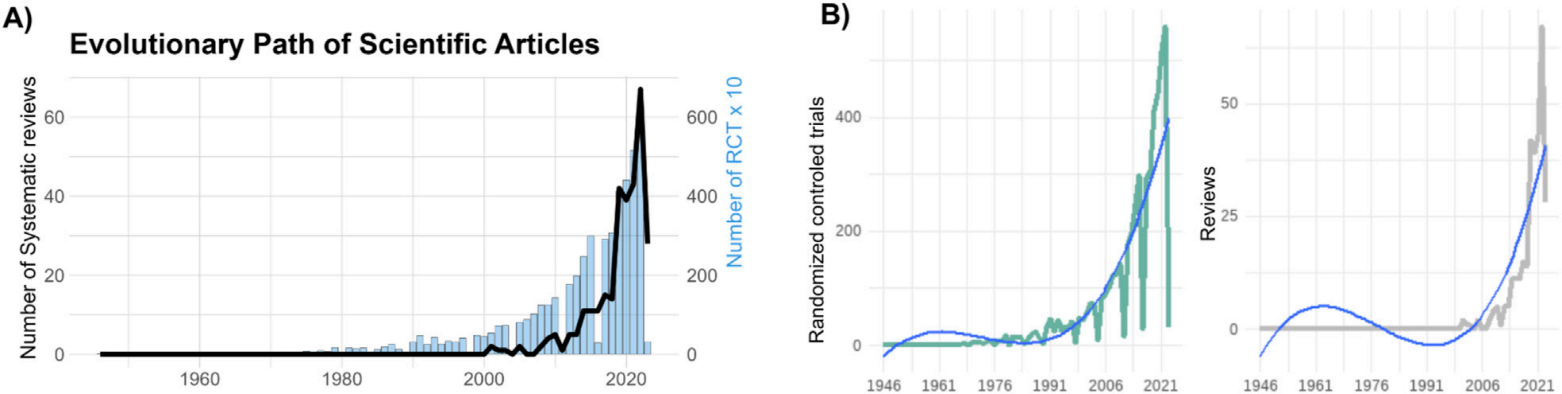
Evolutionary Path of Scientific Articles. **(A)** Temporal evolution of scientific articles in the field of exercise and chronic kidney disease. The graph showcases the growth of systematic reviews, represented by the black line and randomized controlled trials (RCTs), represented as blue bars over different time periods, revealing the dynamic trajectory of knowledge expansion. For a better visualization, we divided by 10 the number of RCTs to be fitted in the same figure. **(B)** Showcases a positive polynomial curve depicting the cumulative number of RCTs over time (left panel), and systematic reviews (right panel). The curve’s upward trajectory accentuates the growth of RCTs investigating the relationship between exercise and chronic kidney disease.

Over time, we observe a substantial growth in both RCTs, and systematic reviews dedicated to studying the impact of exercise on CKD (Figure 3A). Notably, while the number of RCTs significantly surpasses that of systematic reviews, both exhibit a similar upward trend in publication numbers (Figure 3B). The studies included in our analysis span the years 2011–2023, and an interesting pattern emerges. Early studies from 2011 to 2014 primarily explored the effects of exercise without differentiating between exercise modalities. However, a significant shift occurred in 2014, marked by the emergence of studies specifically investigating the effects of aerobic and combined training. Furthermore, from 2018 onwards, more studies have begun to explore a broader range of exercise modalities, encompassing EMS, home-based, isometric, resistance, and respiratory training (Supplementary Figure S1). The primary outcomes assessed in these studies include blood pressure, body composition, cardiorespiratory fitness, functional performance, and quality of life (Supplementary Figure S2). For a more comprehensive understanding of the number and proportion of studies per exercise modality and outcome, please refer to Supplementary Tables S3, S4.

### 3.4 Statistical significance and efficacy of exercise modalities

From 275 reports, 113 (41%) presented an overall P-value <0.05, 68 (24%) demonstrated a *P* < 0.01, 30 (11%) showed a *P* < 10^−3^, and 28 (10%) a *P* < 10^−6^. Notably, 138 (50%) of the reports did not exhibit a statistically significant effect (*P* > 0.05). We observed a higher number of significant meta-analyses (*P* < 0.05) in aerobic and combined training: 18 (15%) and 27 (24%) reports, respectively. Only 4 (3.5%) reports showed a statistically significant effect for EMS training. We found 7 (6.2%) significant reports for home-based training. There were 6 (5.3%) significant reports for isometric training, 10 (8.8%) for resistance training, and 2 (1.8%) for respiratory training. Moreover, all interventions seem to be effective in improving functional performance, except isometric training.

### 3.5 Dialysis vs. non-dialysis

Notably, there is a trend in the included meta-analyses to investigate more ESKD than non-dialysis patients for all interventions included. Regarding the outcomes, 19 (16%) reports with functional performance and cardiorespiratory fitness exhibited a significant P-value. Most outcomes reported in the included meta-analyses investigated ESKD patients, except for body composition (dialysis: *n* = 1/non-dialysis: *n* = 5) and all-cause mortality (dialysis: *n* = 1/non-dialysis: *n* = 2). However, a more comprehensive examination of the robustness of these effects is still required for a clearer understanding.

### 3.6 Analysis of evidence grading

Next, we conducted a comprehensive assessment of our study’s intervention, providing an overview of the statistical significance, heterogeneity, and evidence grading related to various exercise modalities (Table 2). Twenty-eight out of 275 (10%) meta-analyses presented strong or highly suggestive evidence grading for exercise modalities, and thirty-six for outcomes. Aerobic and combined training exhibited the highest number of strong meta-analyses (17% and 22%, respectively) (Table 2). More details about each intervention and outcome are described in Supplementary Table S3, respectively.

**TABLE 2:** Evidence grading of included meta-analyses according to each intervention.

	Total	Aerobic, *n* = 59	Combined, *n* = 67	EMS, *n* = 12	Home-based, *n* = 16	Isometric, *n* = 7	Isotonic^a^, *n* = 97	Resistance, *n* = 27	Respiratory, *n* = 2
*P* value <10^−^6 (*n*, %)	30 (100%)	4 (14%)	7 (25%)	0 (0%)	2 (7.1%)	1 (3.6%)	12 (43%)	1 (3.6%)	1 (3.6%)
*P* value <10^−^3 (*n*, %)	61 (100%)	6 (12%)	5 (10%)	1 (2%)	3 (6%)	4 (8%)	26 (52%)	5 (10%)	0 (0%)
*P* value <0.05 (*n*, %)	38 (100%)	8 (14%)	15 (25%)	3 (5.1%)	2 (3.4%)	1 (1.7%)	25 (42%)	4 (6.8%)	1 (1.7%)
I^2^									
<25%	144 (100%)	26 (17%)	34 (24%)	7 (4.7%)	9 (6.0%)	4 (2.7%)	47 (31%)	15 (10%)	2 (1.3%)
>50%	90 (100%)	25 (27%)	14 (16%)	5 (5.3%)	5 (5.3%)	2 (2.1%)	30 (32%)	9 (9.6%)	0 (0%)
Grading									
Not significant (*n*, %)	146 (100%)	42 (29%)	30 (21%)	9 (6.2%)	9 (6.2%)	1 (0.7%)	38 (26%)	17 (12%)	0 (0%)
Weak (*n*, %)	59 (100%)	8 (13%)	15 (25%)	3 (5.0%)	2 (3.3%)	1 (1.7%)	25 (42%)	4 (6.7%)	1 (1.7%)
Suggestive (*n*, %)	50 (100%)	6 (12%)	5 (10%)	1 (1.9%)	3 (5.8%)	4 (7.7%)	26 (50%)	5 (9.6%)	0 (0%)
Highly suggestive (*n*, %)	5 (100%)	0 (0%)	2 (40%)	0 (0%)	0 (0%)	0 (0%)	3 (50%)	0 (0%)	0 (0%)
Strong (*n*, %)	23 (100%)	4 (17%)	5 (22%)	0 (0%)	2 (8.3%)	1 (4.2%)	9 (38%)	1 (4.2%)	1 (4.2%)

EMS, Eletromyostimulation.

^a^ Isotonic exercises were attributed to all meta-analysis that did not specified or assessed the effect of an specific type of exercise.

### 3.7 Strength of evidence for exercise modalities

**FIGURE 4:**
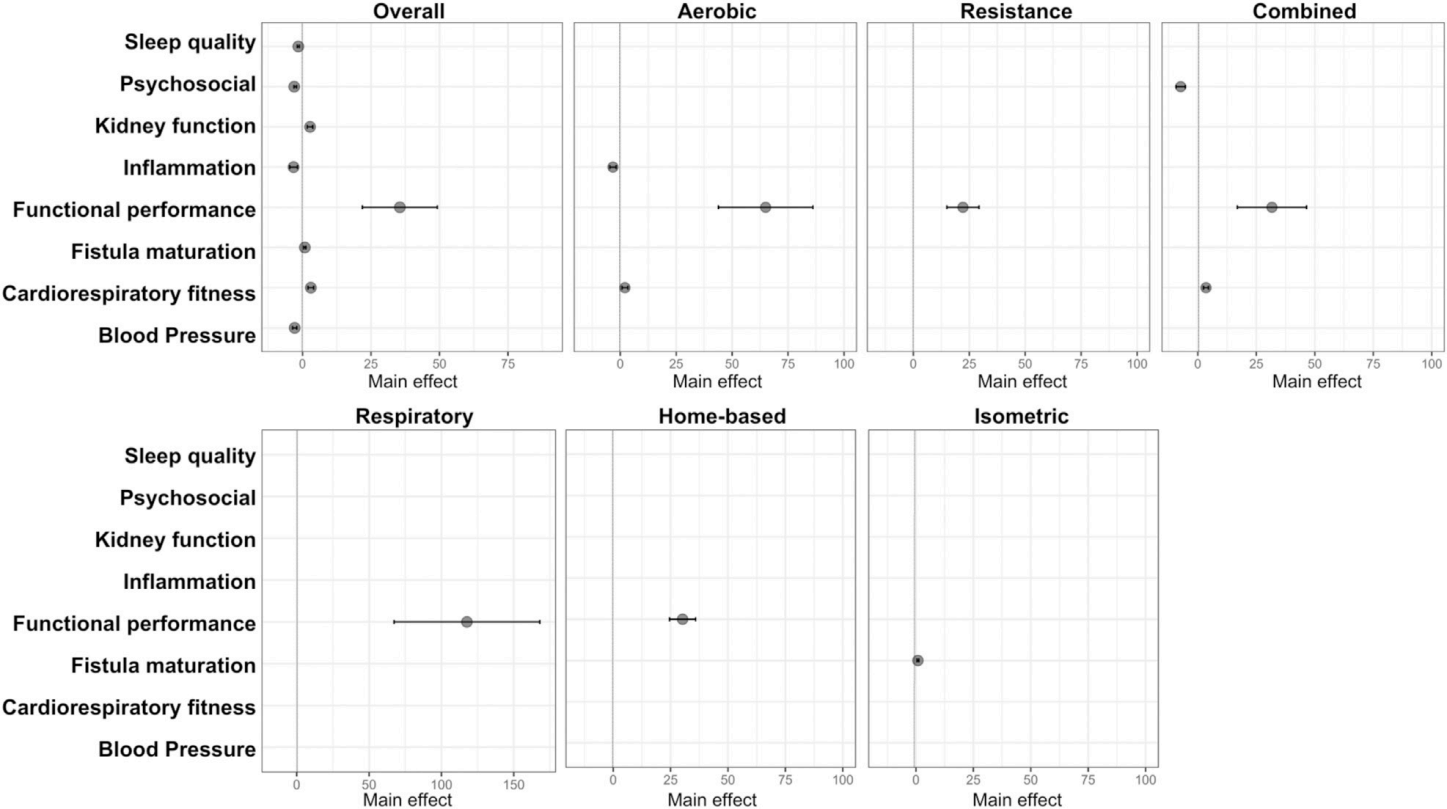
Forest plot displaying results with strong or highly suggestive evidence from the umbrella review on each exercise intervention and improved outcomes in CKD.

After selection of the highly suggestive and strong studies, we performed an assessment of the strength of evidence between exercise modalities and outcomes. Specific findings from different exercise modalities emerge as follows: Aerobic training exhibits robust evidence for reducing inflammatory markers (main effect: −3.28; 95% CI: −4.68 to −1.88), enhancing functional performance (main effect: 64.98; 95% CI: 43.86–86.11), and improving cardiorespiratory fitness (main effect: 2.1; 95% CI: 0.8–3.4). Combined training significantly impacts psychosocial aspects (main effect: −7.3; 95% CI: −9.31 to −53), functional performance (main effect: 31.68; 95% CI: 16.91–46.46), and cardiorespiratory fitness (main effect: 3.4, 95% CI: 2.4–4.6). Resistance training, respiratory training, and home-based training robustly improve functional performance (main effect: 30.23; 95% CI: 24.55–35.92/main effect: 117.62; 95% CI: 67.26–167.99/main effect: 22.18; 95% CI: 15–29.36, respectively). Isometric training exhibits promise in enhancing fistula maturation (main effect: 0.84; 95% CI: 0.45–1.23). Notably, improvements in functional performance are a common thread across most of these interventions. Illustrated in Figure 4.

## 4 Discussion

This umbrella review depicted the potential benefits of exercise for CKD patients following an extensive summarization of systematic reviews with meta-analysis. The pooled 44 studies provided valuable findings in the specificity of exercise modalities in impacting different outcomes in this population (Andrade et al., 2022; Andrade et al., 2019; Baião et al., 2023; Bernier-Jean et al., 2022; Bogataj et al., 2019; Burrai et al., 2021; Cai et al., 2022; Cheema et al., 2014; Chung et al., 2017; Clarkson et al., 2019; Ferrari et al., 2020; Ferreira et al., 2019; Ferreira et al., 2021; Gomes Neto et al., 2018; Hargrove et al., 2021; Huang et al., 2019; Junqué-Jiménez et al., 2022; Liu et al., 2022; Ma et al., 2022; Matsuzawa et al., 2017; Meng et al., 2022; Nakamura et al., 2020; Nantakool et al., 2022; Nantakool et al., 2020; Pei et al., 2019; Pu et al., 2019; Qiu et al., 2017; Sawant et al., 2014; Scapini et al., 2019; Schardong et al., 2020; Sheng et al., 2014; Smart and Steele, 2011; Song et al., 2022; Song et al., 2018; Thompson et al., 2019; Vanden Wyngaert et al., 2018; Villanego et al., 2020; Wang et al., 2022; Wu et al., 2021; Wu et al., 2020; Yang et al., 2017; Yang et al., 2020; Young et al., 2018; Zhang et al., 2019). We found robust evidence on the effects of aerobic exercise in improving inflammatory markers, functional performance, and cardiorespiratory fitness (Ferrari et al., 2020; Pei et al., 2019). Combined training robustly improved psychosocial parameters (depression and anxiety symptoms), functional performance, and cardiorespiratory fitness (Ferrari et al., 2020; Gomes Neto et al., 2018; Huang et al., 2019; Villanego et al., 2020). Home-based, respiratory, and resistance training demonstrated strong evidence in improving functional performance (Ferrari et al., 2020; Gomes Neto et al., 2018; Junqué-Jiménez et al., 2022). Finally, isometric training seems to robustly improve fistula maturation (Nantakool et al., 2022). Although previous and ongoing randomized controlled trials and meta-analyses continue to investigate the impact of different exercise modalities in this population, addressing this in a single study can be impractical. Therefore, this review serves as a valuable resource for enhancing our understanding of the effect of exercise in CKD by summarizing the main results of recent systematic reviews with meta-analysis. This study may act as a guide for future research in this field, shedding some light on how each exercise modality may benefit CKD-related outcomes.

CKD patients often experience mitochondrial dysfunction, strongly associated with oxidative stress, impairing aging process, inflammatory profile, and functional performance (Bai et al., 2023; Rao et al., 2018). On the other hand, aerobic exercise has been shown to stimulate mitochondrial biogenesis, mainly through the activation of peroxisome proliferator-activated receptor gamma coactivator 1-alpha (PGC-1α) (Memme et al., 2021; Alizadeh Pahlavani et al., 2022). This molecular pathway has been recognized as an important signaling pathway to improve oxidative metabolism and overall homeostasis (Bai et al., 2023; Rao et al., 2018; Alizadeh Pahlavani et al., 2022; Koch et al., 2017). Moreover, aerobic training has been found to have anti-inflammatory effects (Petersen and Pedersen, 2005). This is a relevant scenario in the field of nephrology as chronic inflammation is a hallmark of CKD, driven by the upregulation of pro-inflammatory cytokines, such as IL-6 and TNF-α (Ebert et al., 2021). Notably, aerobic exercise can downregulate the expression of these pro-inflammatory cytokines by inhibiting the activation of nuclear factor-kappa B (NF-κB), leading to a better inflammatory profile (Diniz et al., 2021; Guo et al., 2022). This inhibition is partly mediated through the activation of adenosine monophosphate-activated protein kinase (AMPK), a metabolic sensor that also has anti-inflammatory properties (Diniz et al., 2021; Xiang et al., 2019). These molecular mechanisms provide a basis for understanding the robust effects of aerobic exercise observed in inflammation, functional performance, and cardiorespiratory fitness in our review.

Resistance training has been widely used by clinicians and researchers to improve health-related parameters in CKD (Corrêa et al., 2021a; Gadelha et al., 2021; Corrêa et al., 2021b; Deus et al., 2022). This training modality is underpinned by several mechanisms related to the increase in muscle mass and strength, which, in turn, contribute to its robust effects presented by this study. One of the primary mechanisms driving the benefits of resistance training is the promotion of muscle hypertrophy. Resistance training involves subjecting muscles to external resistance, which leads to muscle damage at the microscopic level (Schoenfeld, 2010; Lim et al., 2022). This damage triggers a repair and growth process, primarily promoted by the activation of satellite cells, leading to muscle regeneration and growth (Fukada et al., 2022). In response to resistance training, these satellite cells become activated and fuse with existing muscle fibers, resulting in an increase in muscle size. This process is known as myogenesis.

Moreover, resistance training stimulates the synthesis of muscle proteins, especially myofibrillar proteins (Schoenfeld, 2010; Schiaffino et al., 2013). The mechanistic target of rapamycin (mTOR) pathway plays a central role in this process. mTOR is a signaling pathway that regulates protein synthesis, cell growth, and muscle hypertrophy (Schiaffino et al., 2013). Resistance training activates mTOR, leading to increased protein synthesis and muscle growth. Additionally, resistance training is known to enhance muscle strength in CKD patients (Corrêa et al., 2021a; Gadelha et al., 2021; Corrêa et al., 2021b). This is partly achieved through neural adaptations. The mechanistic insights mentioned above provide a basis for understanding why resistance training robustly improves functional performance. Furthermore, the strong associations between higher muscle mass and a better disease prognosis and survival are rooted in the benefits of resistance training (Gadelha et al., 2021; de Luca Corrêa et al., 2023). Increased muscle mass is associated with improved metabolic health, better glucose control, and a reduced risk of insulin resistance, which might be a key tool to improve the health status from individuals with CKD (Gadelha et al., 2021; Deus et al., 2022; Deus et al., 2021; de Luca Corrêa et al., 2023).

Taken together it is rational to combine aerobic and resistance training to achieve an optimized benefit in this population, as has been explored in previous studies (Andrade et al., 2019; Cai et al., 2022; Ferrari et al., 2020; Ferreira et al., 2021; Gomes Neto et al., 2018; Nakamura et al., 2020; Scapini et al., 2019; Song et al., 2022; Villanego et al., 2020). The synergy of these two modalities, known as combined training, is more likely to induce a comprehensive range of beneficial effects, including those related to psychosocial wellbeing (Ferreira et al., 2021). The mechanisms underlying the effects of combined training may be attributed to the additive or synergistic effects of aerobic and resistance training (Dor-Haim et al., 2018). As discussed earlier, resistance exercises in combined training promote muscle hypertrophy and the synthesis of muscle proteins. This leads to an increase in muscle mass and strength, which are associated with enhanced physical performance and overall health. Aerobic exercises, on the other hand, improve cardiorespiratory fitness and metabolic parameters (Diniz et al., 2021; Guo et al., 2022; Fleg, 2012; Seals et al., 2019). Furthermore, the combination of aerobic and resistance training can have a positive impact on psychological wellbeing.

One of the mechanisms for this effect is the release of endorphins during exercise (Harber and Sutton, 1984; Mikkelsen et al., 2017), playing a key role in improving mood, symptoms of depression, and enhancing overall psychosocial wellbeing (Harber and Sutton, 1984; Mikkelsen et al., 2017). Another mechanism is the modulation of neurotransmitters and hormones, including serotonin and brain-derived neurotrophic factor (BDNF) (Deus et al., 2021). Both aerobic and resistance training have been shown to influence these neurochemicals, which are associated with mood regulation and cognitive function. The approach of combined training, by addressing both physical and psychological aspects, contributes to its consistent effects on psychosocial factors, including reduced depression and anxiety symptoms.

Isometric training involves static muscle contractions in which the length of the muscle remains unchanged (Nantakool et al., 2022; Nantakool et al., 2020). Unlike dynamic exercises such as aerobic or resistance training, isometric exercises focus on maintaining a specific muscle position or tension without movement. One of the key mechanisms through which isometric training can significantly impact fistula maturation is related to its effects on vascular function (Nantakool et al., 2022; Nantakool et al., 2020). Isometric exercises can generate sustained muscle tension and increase intra-arterial pressure in the exercising limb. This elevation in intra-arterial pressure is thought to stimulate adaptive responses in the blood vessels, including the vascular endothelium (Souza et al., 2018). Moreover, isometric training has been shown to enhance endothelial function by promoting the release of nitric oxide, a potent vasodilator molecule (Souza et al., 2018). This vasodilatory effect can improve blood flow, leading to vascular benefits. These mechanisms highlight the relevance of isometric training in improving vascular access and overall CKD management, especially in patients undergoing hemodialysis.

Despite the beneficial effects of exercise, adherence to exercise programs remains a challenge in this field (Clyne and Anding-Rost, 2021). In this context, modalities such as home-based training offer the potential to encourage more participants to continue their exercise routines. Our findings reveal robust evidence supporting the effectiveness of home-based training in improving functional performance. Home-based approaches can accommodate different exercise modalities, tailored to meet individual patient needs (de Araújo et al., 2023; Liu-Ambrose et al., 2019). However, it is essential to note that patients receiving home-based training may be physically distant from the clinical support typically available in hospitals and clinics. As a result, strict adherence to established procedures for monitoring vital signs and assessing adverse events, as outlined in the primary exercise guidelines, is crucial to ensure safety and efficacy. Exercise training should adopt a more conservative approach, and any progressive increase in intensity or volume should be approached with caution. Similarly, respiratory interventions are designed to address specific physiological needs. While its impacts may be less pronounced compared to other modalities (Corrêa et al., 2021a; de Araújo et al., 2023; Corrêa et al., 2021b), it offers valuable benefits, such as enhancing lung capacity, addressing compromised lung function in CKD patients, and contributing to overall mobility and functionality (Slee and Reid, 2022; Wilund et al., 2021). By understanding the contributions of each exercise modality, clinicians and researchers can design targeted exercise interventions that address the diverse needs of CKD patients (Evans et al., 2022). This personalized approach holds the potential to optimize outcomes and enhance the quality of life for individuals grappling with the challenges of CKD.

To attain optimized benefits from physical conditioning through exercise, CKD patients require appropriate care and interventions. These measures are applicable from the early stages of CKD but become crucial for patients in advanced stages (3B and higher) and to those undergoing dialysis treatments. Of utmost importance are the nutritional aspects that require a healthy diet with adequate protein and calorie intake, low sodium content, and often fluid restriction (Ikizler et al., 2020). Additionally, effective control and treatment of diabetes mellitus, blood pressure and metabolic acidosis may also be necessary (Navaneethan et al., 2023; Flythe et al., 2020). The management of bone mineral disorders, especially secondary hyperparathyroidism, is also important and includes the use of phosphate binders and calcimimetics as needed (Ketteler et al., 2018). Anemia, a nearly universal finding in the advanced stages of CKD, is managed through iron replacement and erythropoiesis-stimulating agents. Correcting anemia to recommended hemoglobin target levels is crucial not only for exercise endurance but also for a variety of other benefits (Babitt et al., 2021). Ultimately, having adequate vascular access, preferably through an arteriovenous fistula, is essential for achieving proper dialysis dosing. Without it, none of the aforementioned interventions will be successful (Baker et al., 2022).

This study presents some limitations that should be mentioned. First, the findings from this study rely on published meta-analyses, so we should not assume which exercise modality is the best to improve one outcome of another. However, this study was the first to highlight the main effects of each exercise modality on different outcomes available in the literature. Second, the heterogeneity of individuals with CKD may impact the main findings of this study, and we encourage new meta-analysis addressing specific subpopulations of this conditions, including, sex differences, dialysis modality, and dialysis technique, which were not factors addressed by the included studies. An important limitation of this study is that the included systematic reviews and meta-analyses did not provide specific details about the impact of training features according to age, and years in dialysis vintage, or specific CKD stage. For the latter, studies have broadly classified as either dialysis and non-dialysis groups, which included mostly CKD stages 3–5. Additionally, while all protocols included in this study were chronic (lasting more than 4 weeks), the specific duration of different exercise modalities and the impact on cardio-respiratory fitness and other measured parameters were not consistently reported. Future studies and systematic reviews should consider the inclusion, duration of modality and analysis of these subgroups to provide more detailed and personalized insights into how different exercise modalities may benefit patients at different stages of the disease, age groups, and dialysis durations. The insights garnered from this umbrella review have significant implications for the management of CKD. This study’s findings provide clinicians, researchers, and policymakers with evidence-based guidance for implementing exercise interventions within CKD management protocols. Furthermore, the comprehensive synthesis serves as a steppingstone for future research endeavors, inviting exploration of mechanistic underpinnings of exercise’s impact on CKD. Our study has far-reaching implications for CKD management, influencing clinical practice, research priorities, and patient-centered care. Finally, investigating optimal exercise intensity, frequency, and duration for specific outcomes and CKD stages can refine intervention protocols.

## 5 Conclusion

This umbrella review explored different exercise interventions in CKD, offering insights into the application of exercise as a complementary therapeutic tool for this population. The interplay between exercise modalities and outcomes underscored the potential of exercise in improving CKD patients. In sum, exercise training is a potent non-pharmacological tool to improve CKD-related outcomes in non-dialysis and dialysis patients.
